# GLP-2 Is Locally Produced From Human Islets and Balances Inflammation Through an Inter-Islet-Immune Cell Crosstalk

**DOI:** 10.3389/fendo.2021.697120

**Published:** 2021-07-05

**Authors:** Wei He, Osmond D. Rebello, Antonia Henne, Fabian Nikolka, Thomas Klein, Kathrin Maedler

**Affiliations:** ^1^ Centre for Biomolecular Interactions Bremen, University of Bremen, Bremen, Germany; ^2^ Department of Bioinformatics and Biochemistry and Braunschweig Integrated Center of Systems Biology (BRICS), Technische Universität Braunschweig, Braunschweig, Germany; ^3^ Faculty of Chemistry and Pharmacy, Julius-Maximilians-Universität Würzburg, Würzburg, Germany; ^4^ CardioMetabolic Diseases Research, Boehringer Ingelheim GmbH & Co. KG, Biberach, Germany

**Keywords:** GLP-2, islets, beta-cell, inflammation, diabetes, GLP-2R, alpha-cell

## Abstract

Glucagon-like peptide-1 (GLP-1) shows robust protective effects on β-cell survival and function and GLP-1 based therapies are successfully applied for type-2 diabetes (T2D) and obesity. Another cleavage product of pro-glucagon, Glucagon-like peptide-2 (GLP-2; both GLP-1 and GLP-2 are inactivated by DPP-4) has received little attention in its action inside pancreatic islets. In this study, we investigated GLP-2 production, GLP-2 receptor (GLP-2R) expression and the effect of GLP-2R activation in human islets. Isolated human islets from non-diabetic donors were exposed to diabetogenic conditions: high glucose, palmitate, cytokine mix (IL-1β/IFN-γ) or Lipopolysaccharide (LPS) in the presence or absence of the DPP4-inhibitor linagliptin, the TLR4 inhibitor TAK-242, the GLP-2R agonist teduglutide and/or its antagonist GLP-2(3-33). Human islets under control conditions secreted active GLP-2 (full-length, non-cleaved by DPP4) into the culture media, which was increased by combined high glucose/palmitate, the cytokine mix and LPS and highly potentiated by linagliptin. Low but reproducible GLP-2R mRNA expression was found in all analyzed human islet isolations from 10 donors, which was reduced by pro-inflammatory stimuli: the cytokine mix and LPS. GLP-2R activation by teduglutide neither affected acute or glucose stimulated insulin secretion nor insulin content. Also, teduglutide had no effect on high glucose/palmitate- or LPS-induced dysfunction in cultured human islets but dampened LPS-induced macrophage-dependent *IL1B and IL10* expression, while its antagonist GLP-2(3-33) abolished such reduction. In contrast, the expression of islet macrophage-independent cytokines *IL6*, *IL8* and *TNF* was not affected by teduglutide. Medium conditioned by teduglutide-exposed human islets attenuated M1-like polarization of human monocyte-derived macrophages, evidenced by a lower mRNA expression of pro-inflammatory cytokines, compared to vehicle treated islets, and a reduced production of itaconate and succinate, marker metabolites of pro-inflammatory macrophages. Our results reveal intra-islet production of GLP-2 and GLP-2R expression in human islets. Despite no impact on β-cell function, local GLP-2R activation reduced islet inflammation which might be mediated by a crosstalk between endocrine cells and macrophages.

## Introduction

Proglucagon-derived peptides (PGDPs) are structurally related gut hormones produced from alternative post-translational enzymatic processing of proglucagon, one of them is glucagon produced from α-cells of pancreatic islets, two other ones are the sister peptides glucagon-like peptides 1 and 2 (GLP-1 and -2), all encoded in the proglucagon gene (*GCG*) (1). Stimulated by the ingestion of nutrients, GLP-1 and GLP-2 are co-secreted from the enteroendocrine L cells of the intestine through proglucagon processing by prohormone convertase 1/3 (PC1/3) (1).

Classically, in pancreatic islets, the proglucagon gene is highly expressed in α-cells; it is cleaved by PC2 to produce glucagon in response to lowering blood glucose levels. More recent studies have also revealed pancreatic α-cells’ production of GLP-1 (2–5). In line with this, PC1/3 is expressed in murine and human α-cells (2–6). Due to the nature of co-production of GLP-1 and GLP-2 by PC1/3, it is assumable that GLP-2 may also be locally produced in pancreatic islets.

GLP-1 and GLP-2 exert multiple direct and indirect actions on nutrient processing and energy metabolism (1), whereas they are rapidly degraded by dipeptidyl peptidase-4 (DPP4), an ubiquitously expressed proteolytic enzyme, which cleaves both GLP-1 and GLP-2 into inactive forms (7). Due to GLP-1’s robust potentiation of glucose stimulated insulin secretion, inhibition of glucagon secretion, gastric emptying and promotion of satiety, various DPP4-inhibitors as well as agonists of the glucagon-like peptide-1 receptor (GLP-1R) are established in the therapy of type 2 diabetes (T2D) (8). The GLP-2R agonist teduglutide is approved for treating short bowel syndrome, as GLP-2 promotes intestine growth and nutrient absorption (9). While the beneficial role of intra-islet GLP-1 action has been extensively investigated, the knowledge of local GLP-2 action in islets is very limited.

There is some controversy of the expression of both GLP-1R and GLP-2R, mainly because of the lack of sufficiently sensitive antibodies. While GLP-1R is widely expressed among organs, GLP-2R expression is restricted and was only found and confirmed primarily in enteroendocrine cells and enteric neurons of the GI tract, subepithelial myofibroblasts and in some regions of the central nervous system (CNS) subepithelial myofibroblasts and in some regions of the CNS (9, 10). Lesser GLP-2R protein was found in human and rodent islets and localized to the α-cell with proglucagon colocalization (11). In line with this, GLP-2 acutely stimulates glucagon secretion (11). Consistent but low GLP-2R mRNA was confirmed in rodent α-cell lines as well as in a human β-cell line and in mouse islets (12), the latter being in contrast to a more recent study (13). GLP-2 had no effect on glucose-stimulated insulin secretion (GSIS) in isolated mouse islets (12).

As there is limited knowledge on the impact of GLP-2R activation in human islets, this study was initiated to investigate intra-islet production of GLP-2 and effects of GLP-2R activation in isolated human islets, with a focus on β-cell function and islet inflammation. Studies revealed robust secretion of GLP-2 from human islets with enhanced GLP-2 production by diabetogenic and proinflammatory conditions. However, GLP-2 agonism by the DPP4-resistant agonist teduglutide had no effect on insulin secretion in cultured human islets, whereas it dampened LPS-induced expression of IL-1β and IL-10. Further experiments with islet conditioned medium suggested a GLP-2 mediated protection from a pro-inflammatory crosstalk between endocrine cells and islet macrophages.

## Methods

### Human Islet Isolation, Culture, Treatment, and GLP-2 Analysis

Human islets were isolated from pancreases of non-diabetic organ donors (both males and females) at the Universities of Wisconsin, Lille or ProdoLabs and cultured on Biocoat Collagen I coated dishes (#356400, Corning, ME, USA referred to as extracellular matrix (ECM)). Human islets were cultured in complete CMRL-1066 (Invitrogen) medium at 5.5 mM glucose and exposed to complex diabetogenic conditions: 22.2 mM glucose, 0.5 mM palmitic acid, the mixture of 2 ng/ml recombinant human IL-1β (R&D Systems, Minneapolis, MN) plus 1,000 U/ml recombinant human IFN-γ (Pepro Tech, Rocky Hill, New Jersey) for 72h. To induce maximal TLR4 activation, attached islets were serum-starved for 6h (14) and then treated with the TLR4 ligand lipopolysaccharide (LPS, 20 μg/ml, from *E. coli* O111:B4, Sigma-Aldrich, Steinheim, Germany) for 24h with or without 10 μM TLR4 inhibitor TAK-242 [patent by Takeda Pharmaceutical (Osaka, Japan), synthesized by Servier (Suresnes, France) according to the published chemical structure (15, 16)]. Palmitic acid was dissolved as described previously (17). The DPP4-inhibitor linagliptin (30 nM), or the DPP4-resistant GLP-2 analogue and GLP-2R agonist teduglutide (10 nM) with and without the GLP-2R antagonist GLP-2(3-33) (5 nM, all Biotrend, Cologne, Germany) were added to the respective treatment conditions. Cell culture supernatants were immediately snap-frozen and later analyzed by an inhouse established assay to specifically recognize active GLP-2 as described before (18). All human islet experiments were performed in the islet biology laboratory, University of Bremen, conducted in strict accordance with the World Medical Association’s Declaration of Helsinki as well as with all relevant institutional and national guidelines, with the appropriate institutional ethics committee’s prior approval. The studies involving material from human organ donors were reviewed and approved by the Ethics Committee of the University of Bremen. The study complied with all relevant ethical regulations for work with human cells for research purposes. Organ donors are not identifiable and anonymous, such approved experiments using human islet cells for research is covered by the NIH Exemption 4 (Regulation PHS 398). Human islets were distributed by the two JDRF and NIH supported approved coordination programs in Europe (Islet for Basic Research program; European Consortium for Islet Transplantation ECIT) and in the US (Integrated Islet Distribution Program IIDP) (19).

### Assay for Active GLP-2

For detection of active GLP-2, a Luminex based assay compatible with the Luminex platform was developed by NMI, University of Tübingen, Germany, in collaboration with Boehringer Ingelheim GmbH & Co. KG, Biberach/Riss, Germany. The Peptide sequence (HADGSFSD) in the N-terminus was synthesized as Cys-Doa-Doa (8-amino-3,6-dioxaoctanoic acid) to increase accessibility of the immunogenic peptide containing variants of the primary sequence of interest. Cystein was included to allow immobilization *via* maleimide groups on the activated carrier proteins (BSA and Ovalbumin). Monoclonal and polyclonal antibodies to the above sequence were generated in rats and rabbits, respectively. Cross reactivity to GLP-1 was analyzed using the multiplex Luminex assay platform on a Luminex 100 system in accordance to classical Luminex based assays. Briefly, 20 µl of sample were incubated in 100 µl ELISA blocking buffer (#1112589, Roche Diagnostics GmbH, Mannheim, Germany) in the presence of 20 µl suspension of microparticles (1000 beads linked to Mab to active GLP-2) and incubated overnight at 10°C. After washing 3 times with 100 µl 0.05% Tween 20 in PBS, 40 µl biotinylated detection polyclonal antibody solution (active GLP-2, stock solution 0.1 mg/ml; diluted 1:50 in ELISA blocking buffer) was added and incubated on a shaker for 2h in the dark (22°C). After washing, 40 µl of streptavidin-PE (2 µg/ml diluted 1:500 in ELISA blocking buffer) was added and incubated on a shaker for 30 min. in the dark (22°C). After washing, plate was run on a Luminex 100. Detection limit of active GLP-2 was 20 pg/ml with minimal cross reactivity to GLP-1.

### RNA Extraction and Quantitative RT-PCR Analysis

Total RNA was isolated from cultured human islets with a Trizol extraction system (TriFast, PEQLAB GmbH, Erlangen, Germany), cDNA synthesis and quantitative RT-PCR was performed as previously described (14). The following TaqMan^®^ Gene Expression Assays (Applied Biosystems, Forster City, CA) were used: *IL1B* (Hs01555413_m1), *IL6* (Hs99999032_m1), *TNF* (Hs99999043_m1), *IL8* (Hs00174103_m1), *IL10* (Hs00961622_m1), *PPIA* (Hs99999904_m1).

### Insulin Secretion

Glucose-stimulated insulin secretion (GSIS) was performed as described previously (14). Briefly, human islets were pre-incubated in Krebs-Ringer bicarbonate buffer (KRB) containing 2.8 mM glucose for 30 min, followed by KRB buffer containing 2.8 mM glucose for 1 h (“basal secretion”) and then an additional 1 h in KRB containing 16.7 mM glucose (“stimulated secretion”) with or without the long-acting agonist of the GLP-1 receptor (GLP-1R) exendin-4 (10 nM; Sigma), or teduglutide (10 nM). Islets were washed with PBS and lysed with RIPA lysis buffer to extract total protein, followed by BCA measurement of protein concentration. Secreted insulin and insulin content from total protein were determined with human insulin ELISA kit (ALPCO Diagnostics, Salem, NH), and normalized to total protein concentration.

### M1 Polarization of Human MDMs in Islet-Conditioned Medium

Primary monocytes isolated from human buffy coats using Biocoll^®^ (Bio&SELL, Feucht, Germany) and CD14 microbeads (Miltenyi, Bergisch Gladbach, Germany) were seeded onto tissue culture dishes (1x10e6 cells/ml) in RPMI 1640 medium containing 50 U/ml macrophage colony-stimulating factor (M-CSF; ImmunoTool, Friesoythe, Germany) for 6 days to induce macrophage differentiation. Medium of cultured human islets treated with teduglutide alone or combined with GLP-2(3-33) for 24 h was collected from 3 different islet isolations and these media were pooled and designated as islet-conditioned medium.

Differentiated macrophages were then pre-cultured in islet-conditioned medium 1:4 diluted by standard RPMI 1640 medium for 2 h, followed by addition of 200 ng/ml LPS and 1,000 U/ml IFN-γ for 8 hours to induce M1-like polarization.

### Extraction of Intracellular Metabolites and Total RNA From MDMs

Extraction was performed as previously described (20). Briefly, MDMs on 12-well plates were washed with 0.9% NaCl and quenched with 0.25 ml of -20°C methanol. After adding an equal volume of 4°C deionized water containing 1 µg/ml D_6_-pentanedioic acid (C/D/N Isotopes, Quebec, Canada) as internal standard, cells were disrupted and collected with cell scrapers and transferred to tubes pre-added with 0.25 ml -20°C chloroform. The extracts were vortexed at 1400 rpm for 20 min at 4°C and centrifuged at 17,000 g for 5 min at 4°C. 0.3 ml of the upper aqueous phase was transferred into gas chromatography compatible glass vials and then dried under vacuum at 4°C in CentriVap Concentration System (Labconco, Kansas City, Missouri). The interphase of the cell extracts was collected for total RNA isolation using NucleoSpin^®^ RNA isolation kit (Macherey-Nagel, Düren, Germany).

### GC-MS Measurement of Intracellular Metabolites of MDMs

GC-MS measurement was performed as described (20), using an Agilent 7890B gas chromatograph equipped with a 30 m DB-35ms and 5 m Duraguard capillary column (Agilent, Santa Clara, California) for separation of derivatized metabolites, and an Agilent 5977B MSD system (Agilent) for measurement of metabolites, followed with data processing using the Metabolite Detector software (21).

### Immunofluorescence

Isolated human islets cultured on ECM dishes were fixed in Bouin’s solution for 15 min before embedding in paraffin as previously described (14). 4-µm sections were deparaffinized, rehydrated and incubated overnight at 4°C with primary antibodies against hGLP-2R (1:100; LS-A1312), hGLP-2R (1:100, C36446, both Lifespan Biosciences, Seattle, WA), guinea pig anti-insulin (#A0546, 1:100, Dako, Glostrup, Denmark) or mouse anti-glucagon (#G2654, 1:100, Sigma-Aldrich, Steinheim, Germany), followed by incubation with FITC or Cy3-conjugated secondary antibody (Jackson Immuno Research Laboratories, West Grove, PA) at room temperature for 1h. Slides were mounted with Vectashield with DAPI (Vector Labs, Burlingame, Ca). Fluorescence was analyzed using a Nikon MEA53200 microscope (Nikon GmbH, Dusseldorf, Germany) and images were acquired using NIS-Elements software (Nikon).

### Statistical Analysis

All values were expressed as means ± SEM with the number of independent individual experiments (biological replicates) presented in the figure legends. The different groups were compared by paired two-tail Students t-test (for two groups) or one-way ANOVA with Dunnet’s post-test (for multiple groups) or two-way ANOVA with Sidak’s post-test (for two conditions) as stated in the figure legends. P value<0.05 was considered statistically significant.

## Results

### GLP-2 Secretion From Isolated Human Islets

**Figure 1:**
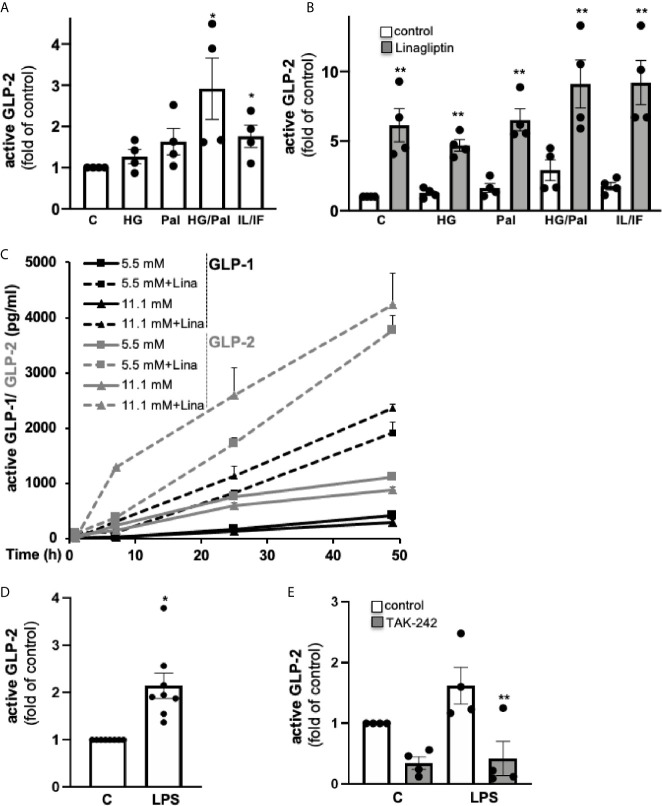
GLP-2 secretion from isolated human islets. Isolated human islets were cultured on ECM-coated dishes **(A–C)** for 72h or during 49 h **(C)** with 5.5 mM glucose (control; C), 22.2 mM glucose (HG), 0.5 mM palmitate (Pal), a combination of HG/Pal or cytokines 2 ng/ml IL-1β/1,000 U/ml IFN-γ (IL/IF), in the absence (control) or presence of 30 nM linagliptin, or **(D, E)** for 24h with 5.5 mM glucose (control; C) or 20 μg/ml LPS in the absence (control) or presence of 10 μM TLR4 inhibitor TAK-242, followed by an ELISA assay of the cell culture supernatants for active GLP-2. Data are normalized to active GLP-2 secretion at untreated control conditions (corresponding to 1.5 ± 0.3 ng/100 islets into the 2 ml culture medium) and shown as means ± SEM from n = 4 **(A, B, E)** or 8 **(D)** different human islet isolations. **(C)** Active GLP-1 and GLP-2 shown as means ± SEM were analyzed from three different islet isolation; data of active GLP-1 are reproduced and thus are transformative from our earlier study (6), where secreted proteins were analyzed during a 49-hour culture of 60 human islets. *p < 0.05 diabetogenic conditions *vs*. control, **p < 0.05 linagliptin or TAK-242 *vs*. vehicle control; by paired two-tailed t-test.

Since GLP-1 and GLP-2 are theoretically co-produced during enzymatic cleavage of proglucagon by PC1/3, one can assume that besides GLP-1, also GLP-2 is locally produced in pancreatic islets. To prove this hypothesis, we cultured human islets isolated from non-diabetic donors for 72h, followed by the analysis of the active form of GLP-2 in the culture medium. The mean concentration of secreted active GLP-2 from four human islet isolations was 1.5 ± 0.3 ng/100 islets (**Figure 1A**). A combined high glucose/palmitate exposure and the mixed pro-inflammatory cytokines IL-1β/IFN-γ increased GLP-2 secretion 2.9- and 1.8-fold, while high glucose or palmitate alone had no significant effect (**Figure 1A**). As expected, linagliptin, a DPP4-inhibitor clinically used for treating T2D, highly increased the amount of active GLP-2 in all conditions (**Figure 1B**), in line with its activity to block cleavage of GLP-2 by DPP4, similar to its effect on GLP-1 secretion from human islets (6). A direct comparison of GLP-1 and GLP-2 from the same islet isolations of our earlier study (6) revealed a similar range of secreted active GLP-1 and active GLP-2 from human islets (**Figure 1C**). We next treated isolated human islets with LPS for 24h, a condition we used previously to induce islet inflammation (22). LPS induced a 2.2-fold increase in active GLP-2 secretion (**Figure 1D**). The TLR4 inhibitor TAK-242 completely abolished such increase, confirming the TLR4-dependent effect of LPS (**Figure 1E**). Our results hereby verify intra-islet production of GLP-2, which is significantly increased by both high glucose/palmitate and by TLR4 activation.

### GLP-2R Expression in Isolated Human Islets

**Figure 2:**
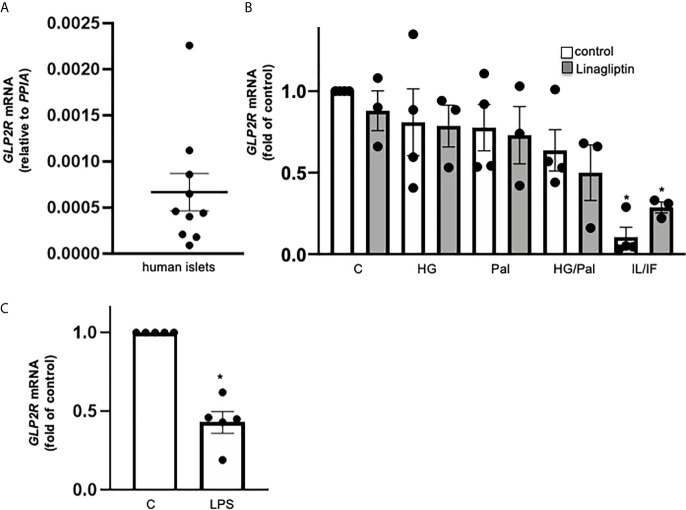
GLP-2R expression in isolated human islets. Isolated human islets were cultured on ECM-coated dishes for 24h **(A, C)** or 72h **(B)** under control medium **(A)** or with 22.2 mM glucose (HG), 0.5 mM palmitate (Pal), or combined HG/Pal or 2 ng/ml IL-1β plus 1,000 U/ml IFN-γ (IL/IF), in the absence (control) or presence of 30 nM linagliptin **(B)** or with 20 μg/ml LPS **(C)**. Total mRNA was isolated and *GLP2R* mRNA analyzed by realtime RT-PCR. Data are shown as means ± SEM from n = 10 **(A)**, 4 **(B)** or 5 **(C)** different human islet isolations. *p < 0.05 diabetogenic conditions *vs*. control by **(B)** 1-way ANOVA with Dunnet’s post-test or **(C)** paired two-tailed t-test.

While there is previous controversy on GLP-2R expression in mouse islets (11–13), little is known in human islets. Therefore, we tested *GLP2R* mRNA levels in isolated human islets from 10 non-diabetic donors. All samples exhibited low but reliable expression of *GLP2R* (Ct 30-35), and the expression levels relative to housekeeping gene *PPIA* ranged from 0.00009 to 0.00226 (**Figure 2A**). Gluco- and lipotoxic treatment conditions (high glucose, palmitate or their combination) had no significant impact on *GLP2R* expression, while highly pro-inflammatory conditions, such as the cytokine mix of IL-1β/IFN-γ (**Figure 2B**) and TLR4 activation by LPS (**Figure 2C**) remarkably reduced *GLP2R* mRNA levels. Linagliptin, which increased active GLP-2, had no significant effect on *GLP2R* expression (**Figure 2B**).

A previous study manifests α-cell localization of GLP-2R in human islets by immunohistochemistry with a non-commercial antibody (11). In contrast, previous elegant scRNA sequencing analyses, could not identify GLP-2R in any of the islet cell types (23, 24). The scRNA sequencing database of human islets (http://hiview.case.edu/public/BetaCellHub/Primaryislet.php) (25, 26) localized expression of *GLP2R* mRNA exclusively in pancreatic stellate cells. Using a previously evaluated commercial antibody to human GLP-2, whose specificity against hGLP-2R has been examined by blocking with its GLP-2R control peptide (27, 28) as well as a second mouse monoclonal antibody to human GLP-2R delivered some GLP-2R co-staining with insulin of very little intensity in isolated human islets from numerous islet isolations (not shown). Because of the low mRNA expression values together with the absent GLP-2R expression from previous RNA-Seq databases, we assume a potential insufficient specificity of these antibodies used by us and others before. While we found *GLP2R* mRNA is reliably but lowly expressed in human islets, future in-depth studies are required to clarify its cellular localization in the human pancreas using highly sensitive and specific analyses.

### Intra-Islet GLR-2R Activation With No Impact on β-Cell Function

Considering the intra-islet GLP-2 secretion and low *GLP2R* expression, we then investigated a possible local action of GLP-2 in human islets on insulin secretion by applying the DPP4-resistant GLP-2R agonist teduglutide (9) with and without the GLP-2R antagonist GLP-2(3-33), which has been shown to antagonize GLP-2R signaling in various cellular and animal models (29), but when applied alone also acts as partial GLP-2R agonist (29, 30). Concentrations were based on previous published studies in GLP-2R expressing cells, rodent and human β-cell lines and human islets, respectively (6, 12, 30, 31).

**Figure 3:**
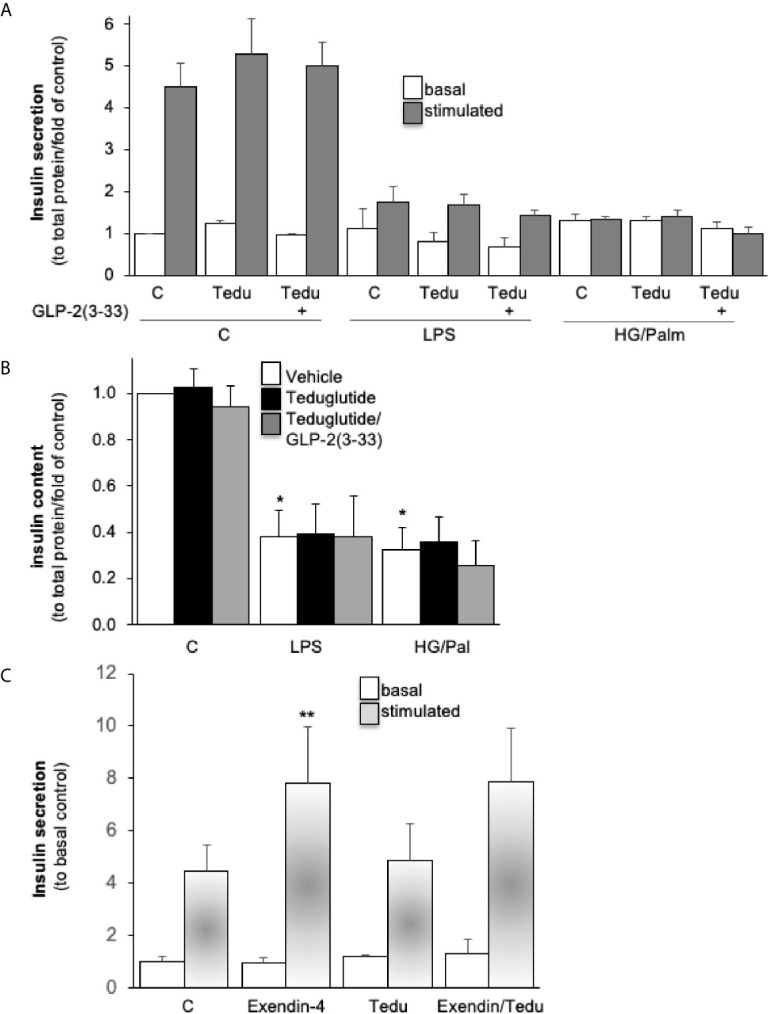
Intra-islet GLR-2R activation with no impact on β-cell function. Isolated human islets were cultured on ECM-coated dishes and pretreated with 10 nM teduglutide or plus 5 nM GLP-2(3-33) for 2 hours, then LPS or a combined HG/Pal was added into the culture medium and maintained for 72h. Afterwards, cultured islets were analyzed for **(A)** glucose-stimulated insulin secretion (2.8mM glucose (basal) for 1 hour, followed by 16.7mM glucose (stimulated) for 1 hour, and **(B)** intracellular insulin content and presented as secreted insulin to total protein content and normalized to the basal control condition **(A, B)**. Acute effect of teduglutide during glucose stimulation; human islets were incubated after the 72h culture at control with 2.8mM glucose (basal) for 1 hour, followed by 16.7mM glucose (stimulated) for 1 hour with the addition of exendin-4, teduglutide or both **(C)**. Data are presented as means ± SEM from 3 **(A, B)** or one **(C)** different human islet isolations. *p < 0.05 LPS/glucose *vs*. vehicle control, **p < 0.05 exendin-4 *vs*. respective control, both under glucose stimulated conditions by paired two-tailed t-test.

Chronic exposure of human islets to classical diabetogenic conditions, such as gluco-/lipotoxicity (high glucose/palmitate) and inflammation (LPS stimulation) was used to impair β-cell function, both diabetogenic conditions completely abolished glucose-stimulated insulin secretion (GSIS, **Figure 3A**) and reduced insulin content (**Figure 3B**), as shown before in numerous studies (14, 32, 33). Neither treatment of teduglutide nor a combined teduglutide and GLP-2(3-33) exhibited any impact on GSIS and insulin content in control, high glucose/palmitate or LPS treated human islets (**Figures 3A, B**). There was also no effect of teduglutide on acute basal and stimulated insulin secretion in human islets (**Figure 3C**). These data are in line with previous analyses in mouse islets (12) and confirm no effect of GLP-2R activation on insulin secretion in both mouse and human islets.

### Intra-Islet GLR-2R Activation Dampened LPS-Induced Islet Inflammation

**Figure 4:**
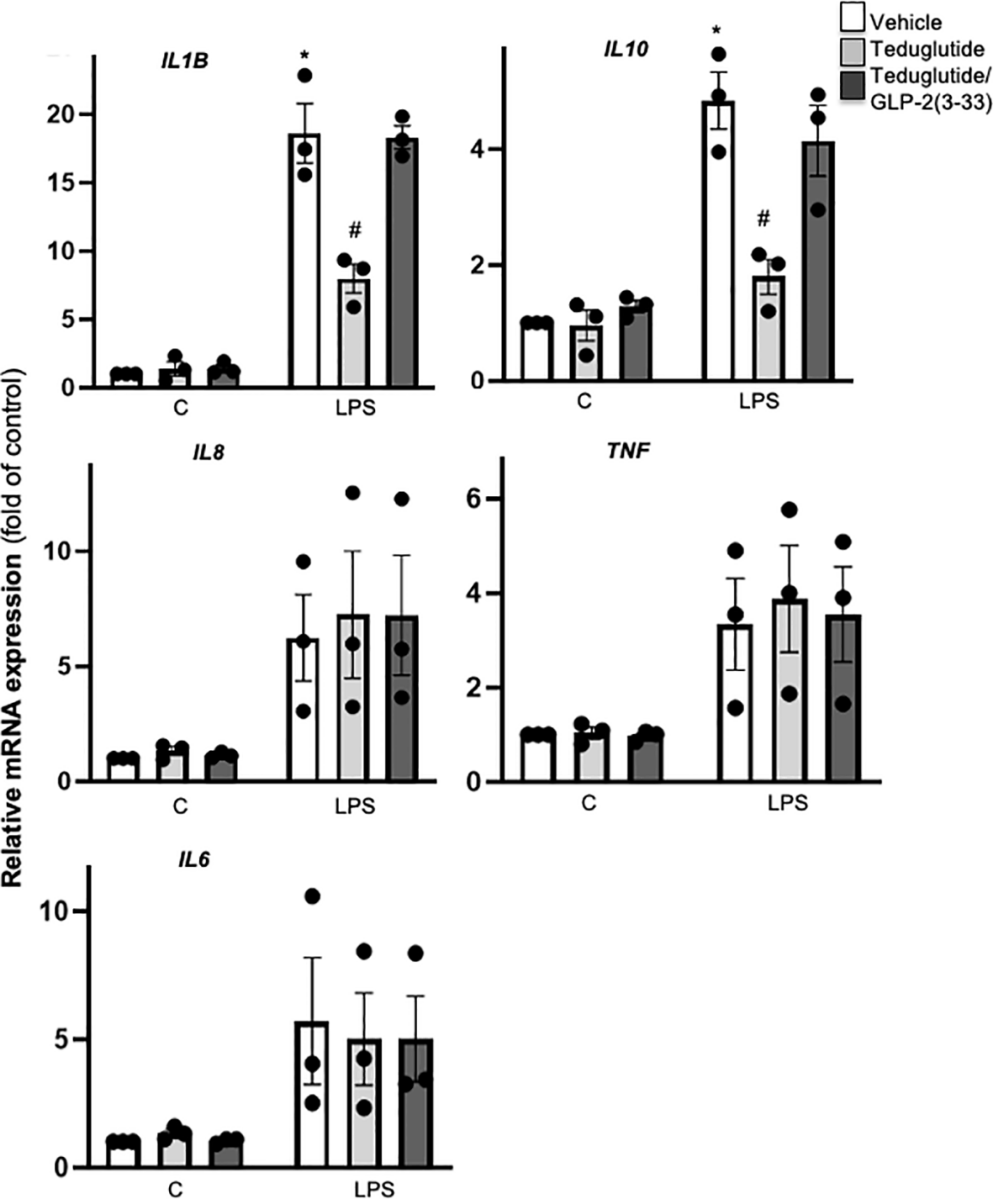
Intra-islet GLR-2R activation dampened LPS-induced islet inflammation. Isolated human islets were cultured on ECM-coated dishes and treated with 10 nM teduglutide or 10 nM teduglutide plus 5 nM GLP-2(3-33) for 2 hours, then LPS was added to the culture medium for 24h. Total RNA was isolated and cytokine and chemokine expression analyzed by realtime RT-PCR. Data are shown as means ± SEM from n = 3 different human islet isolations. *p < 0.05 *vs*. control, ^#^p < 0.05 teduglutide *vs*. vehicle control by 2-way ANOVA with Sidak’s post-test.

GLP-2R activation ameliorates intestinal and hepatic inflammation in various animal models (34–39). Despite no effect on β-cell function, we reasoned whether GLP-2R activation alters islet inflammation. Using our established LPS stimulation protocol in cultured human islets (14), we observed a significant LPS induced stimulation of *IL1B*, *IL10* mRNA as well as a trend towards increased *IL6, IL8 and TNF* mRNA. LPS-stimulated *IL1B* and *IL10* expression was attenuated by teduglutide and reversed by the combined exposure of the islets to teduglutide and GLP-2(3-33), indicating an GLP-2R dependent effect (**Figure 4**). In contrast to *IL1B* and *IL10* expression, GLP-2R activation had no effect on the mRNA expression levels of *IL8, TNF* and *IL6* (**Figure 4**).

The difference between these two distinct GLP-2R effects is that TLR4 activated *IL1B* and *IL10* expression in human islets comes by a majority from islet-residing macrophages, while *IL8, TNF* and *IL6* are produced by islet endocrine cells, independent of the presence of macrophages (22, 40). Nevertheless, neither human peripheral blood mononuclear cells (PBMCs) nor any types of human monocytes or macrophages express *GLP2R* (https://www.proteinatlas.org/ENSG00000065325-GLP2R), which we confirmed by qRT-PCR (data not shown). Therefore, we could exclude a direct anti-inflammatory effect of teduglutide, neither on macrophages (no *GLP2R* expression) nor on endocrine cells (no anti-inflammatory effect) in human islets upon TLR4 activation.

### Conditioned Medium From Teduglutide-Treated Islets Attenuated M1 Polarization of Human Macrophages

**Figure 5:**
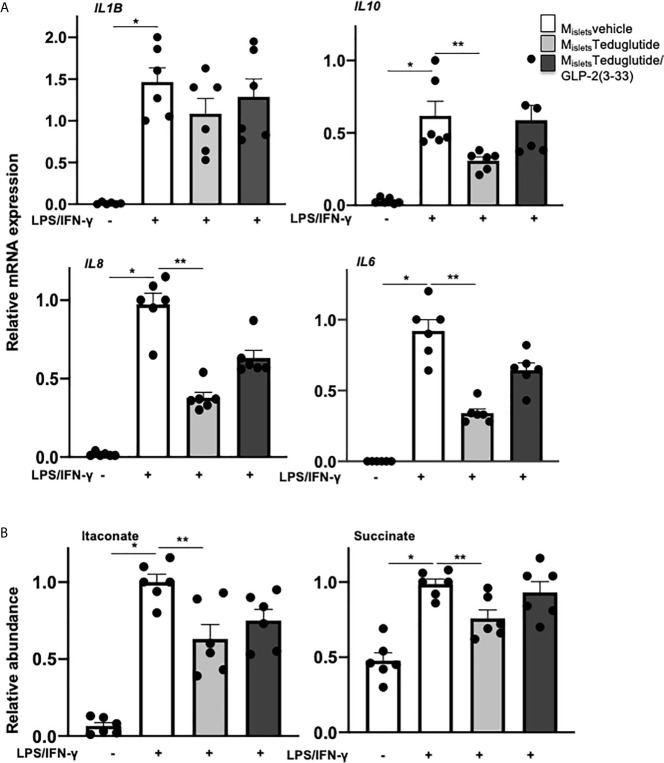
Conditioned medium from teduglutide-treated islets attenuated M1 polarization of human macrophages. Primary human monocyte-derived macrophages (by M-CSF) were cultured in medium pre-conditioned by cultured untreated human islets (M_islets_vehicle) or islets treated with teduglutide alone (M_islets_Teduglutide) or combined with GLP-2(3-33) (M_islets_Teduglutide/GLP-2(3-33)) for 2h, then LPS/IFN-γ was added to the culture and maintained for 8h to induce M1-like polarization, followed by **(A)** total RNA isolation and cytokine expression analysis by realtime RT-PCR and **(B)** intracellular metabolite extraction and measurement by GC-MS. Data are presented as means ± SEM from n=6 biological replicates pooled from two independent experiments of two different macrophage preparations. *p < 0.05 LPS/IFN-γ *vs*. control, **p < 0.05 M_islets_Teduglutide *vs*. M_islets_vehicle LPS/IFN-γ by 1-way ANOVA with Dunnet’s post-test.

Consequently, we speculated that the modulatory effect of LPS-induced islet inflammation may come from a crosstalk between GLP-2 responsive islet cells and islet resident macrophages. To test this, we designed a co-culture experiment using conditioned medium from teduglutide and teduglutide/GLP-2(3-33) treated human islets, similarly as described before (14, 22). Medium conditioned by cultured untreated human islets (M_islets_vehicle), islets treated with teduglutide alone (M_islets_Teduglutide) or combined with GLP-2(3-33) (M_islets_Teduglutide/GLP-2(3-33)) were applied to human monocytes derived macrophages (MDMs) during M1-like polarization stimulated by a combination of LPS and IFN-γ. Gene expression analysis of M1-like macrophages confirmed LPS+IFN-γ induced cytokines and chemokines (**Figure 5A**)*. IL6, IL8* and *IL10* expression was significantly reduced in M1-like macrophages co-cultured with medium from teduglutide treated islets (**Figure 5A**), whereas this phenomenon was abolished by the co-culture medium from human islets treated with the combination of both teduglutide and GLP-2(3-33).

In addition to commonly used cytokines and chemokines as macrophage activation markers, the intracellular metabolite itaconate and succinate are emerging markers recently implicated in pro-inflammatory macrophage polarization (41–43). To further validate the effect of conditioned medium during M1-like macrophage polarization, we measured the intracellular metabolites of these M1-like MDMs by GC-MS. Both itaconate and succinate were highly increased by LPS+IFN-γ on M1-like macrophages, compared to vehicle treatment. Co-culture of conditioned medium from human islets treated with teduglutide but not with the combination of teduglutide with GLP-2(3-33) decreased the accumulation of both itaconate and succinate in M1-like MDMs (**Figure 5B**), suggesting a broad effect of GLP-2R activation to dampen proinflammatory polarization of macrophages. These results support a cell contact-independent paracrine crosstalk between GLP-2 responsive cells and islet macrophages, which contribute to the amelioration of islet inflammation.

### Discussion

In the year 2021, when we celebrate the 100s anniversary of the discovery of insulin, it is just natural that the breakthrough of another family of secreted peptides of common origin from the proglucagon gene: GLP-1, GLP-2 and glucose-dependent insulinotropic polypeptide (GIP) with major implications on the therapy of diabetes was recognized and awarded (44). While the beneficial role of GLP-1 on pancreatic β-cell survival and function has been extensively studied and well understood, knowledge of GLP-2 in islets is scarce. As α-cells secrete GLP-1 (2–5) and GLP-1 and GLP-2 are theoretically co-produced through PC1/3-dependent post-translational proglucagon processing, it is assumable that GLP-2 may also be locally produced in pancreatic islets. In line with this speculation, an ELISA assay specific for detecting active GLP-2 exhibited GLP-2 secretion from cultured human islets which was enhanced by pro-inflammatory diabetogenic conditions. α-cell production of PC1/3 and GLP-1 as well as total GLP-1 secretion from human islets are enhanced under situations of β-cell loss glucotoxicity, lipotoxicity and proinflammatory cytokine exposure (5, 6, 45). The pro-inflammatory cytokine IL-6 promotes α-cell-derived GLP-1 production (2), and LPS induces intra-islet secretion of IL-6 (14). Hence, it is possible that IL-6 may mediate the enhanced GLP-2 secretion in LPS-treated human islets. Increased GLP-2 secretion together with a reduced GLP-2R expression under the pro-inflammatory stimulation by LPS and the cytokines IL-1β/IFNγ observed in this study further assume GLP-2’s potential role during islet inflammation.

GLP-2R is a G protein–coupled receptor (GPCR) responsible for the GLP-2 action (31). It is highly specific for GLP-2 and has only weak affinity to structurally related peptides such as glucagon, GLP-1, and GIP (46). Previous RNASeq analyses did not identify *GLP2R* expression in human islets (23, 24), while it was found in rat islets by transcriptomics but not proteomics analysis (47), indicating a very low expression of *GLP2R*. This is in line with several elegant peptidomics analyses in human islets, e.g (48–50), which could not identify GLP-2R on the protein level. Using specific membrane extraction methods for protein profiling of human islets it was difficult to identify low abundant proteins; and neither GLP-1R nor GLP-2R were found (51). In addition to the nature of its expression, there is further discrepancy regarding GLP2-R’s cellular localization (11, 12) in human and rodent islets. Because of their cross reactivity, the specificity issue of various antibodies against glucagon related peptides is well-known; including anti-GLP-2R antibodies, which are insufficient in both sensitivity and specificity (29). Thus, even by using previously evaluated antibodies (27, 28), we cannot be sure of the cellular localization in human islets. Two data sets (25, 26) identify GLP-2R in human pancreatic stellate cells (PSCs), which is in line with its expression in human and mouse hepatic stellate cells (HSCs) (52). PSCs are multifunctional in the endocrine, where they exist to a small amount of 3% (24), as well as the exocrine pancreas and are thought to contribute to the inflammatory response as well as to tissue regeneration. Although functionally different, both PSCs and HSCs have almost identical mRNA expression profiles (53) including inflammatory cytokines and chemokines analyzed in the present study, such as IL-1, IL-6, TNF and IL-8 as well as TLRs. PSC produced IL-8 is well-known to participate in recruiting inflammatory cells and fostering inflammation in the pancreas (53). Importantly and elegantly shown, loss in GLP-2R signals in hepatic stellate cells also contributes to increased hepatic inflammation (52).

Many physiological studies found an anti-inflammatory role of GLP-2 in the gastrointestinal tract. GLP-2 administration exhibits prominent anti-inflammatory effects in intestinal mucosa of various murine models of colitis (34, 35, 38, 39) and in a postoperative ileus mouse model (37). *In vitro* studies at the cellular level are rare, partly due to the fact that GLP-2R is absent in immune cells. In our current *ex vivo* study, GLP-2R activation by teduglutide reduced LPS-induced *IL1B* and *IL10* expression in human islets. Both cytokines are dependent on islet-associated macrophages (22, 40), whereas macrophages do not express GLP-2R. A plausible speculation would be a crosstalk between GLP-2 responsive intra-islet cells and islet resident macrophages. To support this, primary human macrophages cultured with conditioned medium from teduglutide-treated human islets display dampened M1 polarization, specifically seen by the reduced *IL6*, *IL8* and *IL10* expression. Pro-inflammatory macrophage activation is associated with reprogrammed intracellular metabolism, featuring accumulation of itaconate and succinate (41–43). Both metabolites were reduced in the M1 macrophages cultured with the teduglutide-treated islet conditioned medium, implying that a GLP-2 induced intra-islet crosstalk broadly affects M1 macrophage polarization, rather than simply influences cytokine expression. In *ex vivo* experiments in human islets, teduglutide was able to reduce macrophage dependent *IL1B* expression, which did not happen in primary MDMs cultured with conditioned medium, despite repetitive efforts and parallel analyses of *IL6*, *IL8* and *IL10*, which were indeed reduced by teduglutide-treated islet medium. The different macrophage origin in this experimental setting [islet resident macrophages mainly originate during embryogenesis while primary monocytes are from adult hematogenesis (54, 55)], as well as the islet microenvironment may be accountable for such discrepancy. The pancreatic islet microenvironment is also known to shape a unique phenotype of resident macrophages (56). Although islet conditioned medium contains islet microenvironment-derived soluble factors, temporal effects of intra-islet crosstalk cannot be recapitulated. Nevertheless, GLP-2’s anti-inflammatory action in the intestine, which is mediated by a crosstalk in which GLP-2R expressing enteric neurons secrete vasoactive intestinal polypeptides (VIPs) which in turn elicit anti-inflammatory effects on intestinal immune cells (35) would also support an intra-islet crosstalk triggered by GLP-2 action, in which GLP-2R activation is able to reduce macrophage dependent cytokine expression within human islets. Thus, it seems a common way for GLP-2 to exert anti-inflammatory effects through a crosstalk between GLP-2 responsive cells, which do express GLP-2R and immune cells, which do not express GLP-2R. Macrophage derived inflammatory factors are prominently targeted, as shown in our study, where macrophage dependent *IL1B* and *IL10* expressions are reduced by GLP-2R agonism, but not endocrine cell dependent *IL8, TNF and IL6.* Such has been confirmed by direct MDM analysis, where *IL6* and *IL8* mRNA was also reduced by teduglutide exposed islets.

In terms of glucose homeostasis, current knowledge does not indicate a direct GLP-2 effect, either through acute administration or chronic effects. GLP-2R activation neither affected insulin secretion in isolated human islets in this study, nor had an effect in mouse islets, but increases glucagon secretion in rat pancreata (11, 12). *In vivo* studies also indicate no direct impact of GLP-2R signaling on glucose homeostasis by either acute GLP-2 administration or chronically in GLP-2R-deficient mice (12, 57, 58). Therefore, in contrast to its co-product GLP-1, GLP-2 is unlikely to directly regulate glucose homeostasis.

Considering GLP-2’s anti-inflammatory action, it is conceivable that activation of GLP-2R and its downstream signaling dampen chronic inflammation and hereby protect β-cells. Therefore, future studies should focus on the effect of GLP-2 in a context of a long-term diabetogenic scenario.

In summary, we have revealed intra-islet production of GLP-2 and confirmed GLP-2R expression in human islets. Local activation of GLP-2R had no impact on β-cell function but attenuates islet inflammation by reducing the local activation of pro-inflammatory macrophages. Much further in-depth research is needed to understand GLP-2’s action in the pancreas and the physiological role of secreted GLP-2 and its consequent degradation by DPP4.

## Data Availability Statement

The raw data supporting the conclusions of this article will be made available by the authors, without undue reservation.

## Ethics Statement

Ethical approval for the use of human islets had been granted by the Ethics Committee of the University of Bremen. The study complied with all relevant ethical regulations for work with human cells for research purposes. Written informed consent for participation was not required for this study in accordance with the national legislation and the institutional requirements. Organ donors are not identifiable and anonymous, such approved experiments using human islet cells for research is covered by the NIH Exemption 4 (Regulation PHS 398).

## Author Contributions

Conceptualization: KM, WH, TK. Methodology and analysis: KM (**Figures 1** **–** **4**), WH (**Figures 1** **–** **5**), OR (**Figures 1** **–** **3**), AH, FN (**Figure 5**). Manuscript writing and editing: KM, WH, TK. Resources: KM, TK. Funding acquisition: KM, TK, Supervision: KM, WH, TK. All authors contributed to the article and approved the submitted version.

## Funding

This work was supported by JDRF and the German Research Foundation (DFG). Human pancreatic islets were kindly provided by the NIDDK-funded Integrated Islet Distribution Program (IIDP) at City of Hope, NIH Grant # 2UC4DK098085, the JDRF-funded IIDP Islet Award Initiative and through the ECIT Islet for Basic Research program supported by JDRF (JDRF award 31-2008-413).

## Conflict of Interest

TK is an employee of Boehringer Ingelheim Pharma. Linagliptin is a Boehringer Ingelheim Pharma product.

The remaining authors declare that the research was conducted in the absence of any commercial or financial relationships that could be construed as a potential conflict of interest.
